# The Roles of Protein Tyrosine Phosphatases in Hepatocellular Carcinoma

**DOI:** 10.3390/cancers10030082

**Published:** 2018-03-20

**Authors:** Yide Huang, Yafei Zhang, Lilin Ge, Yao Lin, Hang Fai Kwok

**Affiliations:** 1 Provincial University Key Laboratory of Cellular Stress Response and Metabolic Regulation, College of Life Sciences, Fujian Normal University, Fuzhou 350117, China; ydhuang@fjnu.edu.cn (Y.H.); yafei1zhang@163.com (Y.Z.); 2 Faculty of Health Sciences, University of Macau, Avenida de Universidade, Taipa, Macau, China; gelilin@njucm.edu.cn; 3 Jiangsu Key Laboratory for Functional Substance of Chinese Medicine, School of Pharmacy, Nanjing University of Chinese Medicine, Nanjing 210023, China

**Keywords:** protein-tyrosine phosphatase inhibitors, hepatocellular carcinoma, signaling pathways, therapeutic targets

## Abstract

The protein tyrosine phosphatase (PTP) family is involved in multiple cellular functions and plays an important role in various pathological and physiological processes. In many chronic diseases, for example cancer, PTP is a potential therapeutic target for cancer treatment. In the last two decades, dozens of PTP inhibitors which specifically target individual PTP molecules were developed as therapeutic agents. Hepatocellular carcinoma (HCC) is one of the most common malignant tumors and is the second most lethal cancer worldwide due to a lack of effective therapies. Recent studies have unveiled both oncogenic and tumor suppressive functions of PTP in HCC. Here, we review the current knowledge on the involvement of PTP in HCC and further discuss the possibility of targeting PTP in HCC.

## 1. Introduction

Hepatocellular carcinoma (HCC) is the fifth most common cancer worldwide and ranks second as a cause of cancer mortality [1]. Although liver transplantation and/or surgical resection are the most common and effective treatments for this disease, they still can only be used to treat early-stage HCC. Current therapeutic options for advanced HCC are still limited, and the overall survival rate of advanced HCC patients remains poor because of tumor invasiveness, metastasis, recurrence and resistance to chemotherapy [2]. In order to develop novel and effective therapeutic strategies against HCC, a better understanding of the molecular mechanisms of HCC development and progression is mandatory. Many signaling pathways which involved in cell proliferation, migration, apoptosis and invasion were found to participate in the development of HCC. The accelerated fibrosarcoma/mitogen-activated protein kinase/extracellular-signal-regulated kinase (Raf/MEK/ERK) pathway is one of the key oncogenic pathways involved in the development of HCC. Platelet derived growth factor receptor (PDGFR) and vascular endothelial growth factor receptor (VEGFR) are the two receptors that play an important role in activation of the Raf/MEK/ERK cascade. On the other hand, the signal transducer and activator of transcription 3 (STAT3) pathway is another key pathway participated in the development of HCC. Aberrant STAT3 activation in HCC tumor tissue is associated with proliferation, metastasis and invasion. In addition, over-activation of phosphatidylinositol-3-OH kinase/protein kinase B/mammalian target of rapamycin (PI3K/AKT/mTOR) signaling pathway leads to enhanced proliferation and metastasis in HCC [3,4].

Protein tyrosine phosphorylation plays a central role in cellular physiology and in diseases including cancer [5]. The elaborated balance of tyrosine phosphorylation of proteins in cell is controlled reciprocally by both protein tyrosine kinases (PTKs) and protein tyrosine phosphatases (PTPs). PTKs are responsible for the phosphorylation of tyrosine residues in proteins. In contrast, PTPs remove phosphate at tyrosine residues from phosphorylated proteins. Tyrosine phosphorylation is required for the activation of signaling pathways involved in multiple cellular processes such as proliferation, adhesion and migration. Studies have shown that disturbance of the balance between PTKs and PTPs plays a critical role in the pathogenesis of HCC. In this review, we will focus on the role of different PTPs in the development of HCC and further discuss their importance as a therapeutic target for the treatment of HCC. 

## 2. PTPs Involved in HCC

**Figure 1:**
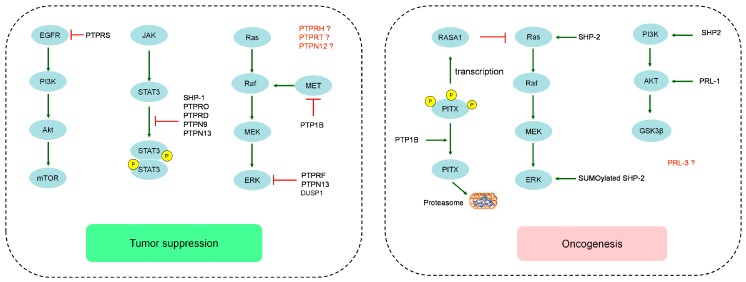
Schematic overview of the role of protein tyrosine phosphatases (PTPs) in hepatocellular carcinoma (HCC). EGFR: epidermal growth factor receptor; PI3K: phosphotidylinsitol-3-OH kinase; Akt: alpha serine/threonine-protein kinase; Mtor: mechanistic target of rapamycin; JAK: janus kinase; STAT3: signal transducer and activator of transcription-3; SHP: Src homology 2-containing phosphotyrosine phosphatase; PTPRO: protein tyrosine phosphatase receptor type O; PTPN: protein tyrosine phosphatase non-receptor; Ras: rat sarcoma; Raf: Rapidly accelerating fibrosarcoma; MEK: MAP kinase kinases; ERK: extracellular signal-regulated kinase; MET: mesenchymal-epithelial transition factor; PTP1B: protein tyrosine phosphatase 1B; PTPRF: protein tyrosine phosphatase receptor type F; DUSP1: dual specificity phosphatase 1; RASA1: Ras p21 protein activator 1; PITX: pituitary homeobox; PRL-1: phosphatase of regenerating liver-1; GSK3β: glycogen synthase kinase 3β.

PTPs consist of a large protein superfamily with 107 members that can be divided into four families—Class I, Class II, Class III and Class IV—according to differences in the amino acid sequence of their catalytic domains [6]. The catalytic residues of Class I, II and III are cysteine, and the catalytic residue of Class IV is aspartate. The Class I PTPs are the largest family with 99 members, which can be further divided into the classical PTPs (38 members) including the receptor PTPs (PTPR) (21 members) and non-receptor PTPs (PTPN) (17 members), and the dual specificity phosphatases (DUSPs) (61 members) capable of dephosphorylating serine, threonine and tyrosine residues. Class II PTPs only contains the low molecular weight phosphatase (LMWPTP) at present. Class III PTPs contain three tyrosine/theronine specific phosphatases (CDC25 A, B and C). Class IV PTPs comprises four tyrosine and serine/tyrosine phosphatases (Eya1, Eya2, Eya3 and Eya4). So far, only 16 PTPs, all belonging to Class I PTPs, were reported to participate in the development or progression of HCC (summarized in Table 1). Most of these PTPs act as tumor suppressors in HCC. But some PTPs such as Src homology 2-containing phosphotyrosine phosphatase 2 (SHP-2) and protein tyrosine phosphatase 1B (PTP1B) can also act as oncogenes depending on the stages of HCC or presence of their differential interacting partners (Figure 1).

### 2.1. The Role of Classical Receptor PTPs in Hepatocellular Carcinoma

Protein tyrosine phosphatase receptor delta(PTPRD), also known as PTPδ, is composed of an extracellular region which contains three Ig-like and eight fibronectin type III-like domains, a single transmembrane segment and two tandem cytoplasmic catalytic domains. Tumor suppressive properties of PTPRD were reported in several human tumors such as glioblastoma multiforme [29,30], melanoma [30], gastric adenocarcinoma [31], lung cancer [32], neuroblastoma [33], and laryngeal squamous cell carcinoma [34]. PTPRD was first suggested as a tumor suppressor in HCC by Urushibara et al. whose results showed selective reduction of PTPRD mRNAs in HepG2 cell line and chemically-induced rat primary hepatoma tissue [35]. It was also reported that PTPRD expression was found to be down-regulated or even completely absent in human HCC cell lines and tumor tissues [7,8]. Patients with high expression of PTPRD have better long-term survival rate and less chance of liver cancer recurrence [7]. The reduction of PTPRD expression in HCC is probably due to DNA methylation as the promoter of PTPRD was found hypermethylated and 5-AzaC and/or trichostatin A (TSA) treatment restored PTPRD expression [8]. In addition to promoter hypermethylation, deletion and mutation of *PTPRD* gene were also identified in HCC cell lines and tumor tissues [30,36]. In hepatitis C virus (HCV)-infected liver tissues, PTPRD expression is impaired due to up-regulation of miR-135a-5p targeting *PTPRD* mRNA, resulting in activation of STAT3 signaling and hepatocarciogenesis [7]. 

Protein tyrosine phosphatase receptor type F (PTPRF) is also known as leukocyte common antigen-related (LAR) and composed of an extracellular domain containing three immunoglobulin domains and eight fibronectin type III domains, a single transmembrane region and two tandem cytoplasmic catalytic domains D1 and D2. PTPRF was frequently down-regulated in HCC patients, and up-regulation of PTPRF was associated with better prognosis [9]. Using a loss-of-function screening of phosphatome to identify genes suppressing tumor initiation in HCC, PTPRF was characterized as one of the top-scoring tumor suppressor candidates. PTPRF suppressed cell proliferation and colony formation in Huh7 and SK-Hep1 cells and inhibited HepG2 xenograft tumor growth in nude mice. The phosphatase activity of PTPRF is required for its tumor suppressor function. PTPRF knockdown led to enhanced phosphorylation of extracellular signal-regulated kinase 1 (ERK1) (Y204) and extracellular signal-regulated kinase 2 (ERK2) (Y187), as PTPRF can directly interact with the upstream factors of ERK v-src avian sarcoma viral oncogene homolog (SRC) and protein phosphatase 2A(PP2A), removing phosphate group from SRC at Y416 and PP2AC (the catalytic subunit C of PP2A) at Y307, leading to suppression of SRC activity and activation of PP2A [9].

Protein tyrosine phosphatase receptor type H (PTPRH), also named stomach cancer-associated protein tyrosine phosphatase-1 (SAP-1), was first identified as a receptor PTP in the human stomach cancer cell line KATO-III [37]. The structure of PTPRH is composed of an extracellular region containing eight fibronectin type III-like repeats and multiple N-glycosylation sites, a single transmembrane region and a single intracellular catalytic domain. Noguchi and colleagues showed that PTPRH inhibits cell proliferation by suppressing growth factor-elicited mitogenic signaling and inducing apoptotic cell death [38]. Investigation in HCC specimens revealed that PTPRH expression in moderately differentiated HCCs and in all poorly differentiated HCCs was greatly reduced or lost compared with that in the adjacent tissues. Overexpression of PTPRH in highly motile human HCC cell lines (HLF and HLE) resulted in a change in cell morphology and remarkable reduction of both migratory activity and growth rate of the cells [10]. These results indicated that PTPRH may play an important role in the progression of HCC, but the underlying mechanism is not yet clear.

Protein tyrosine phosphatase receptor type O (PTPRO), also known as glomerular epithelial protein 1 (GLEPP1), was first discovered in the human renal glomerulus [39]. The structure of PTPRO is composed of a single intracellular catalytic domain that catalyzes the dephosphorylation of tyrosine peptides and a transmembrane domain. PTPRO was found to act as a tumor suppressor in human cancers such as chronic lymphocytic leukemia [40], lung [41], and breast cancer [42]. In HCC, PTPRO expression was significantly reduced in the tumor specimens compared with adjacent tissue [11,43], which is probably caused by hypermethylation on PTPRO promoter. In a rat model of HCC induced by folate/methyl deficient diet (FMD), PTPRO mRNA was significantly reduced and its gene was found hypermethylated at the site located immediate upstream of the transcription start in a genome wide screen for hypermethylated genes. However, PTPRO gene was methylation free in the livers of animals on normal diet. Expression of PTPRO mRNA was confirmed after the transplanted hepatoma was treating with 5-azacytidine. In addition, an investigation on human HCC samples also showed that the CpG island of PTPRO is significantly hypermethylated [43]. The cell proliferation was inhibited and apoptosis was promoted in PTPRO overexpressing HCC cell lines, and tumor number and size were enhanced in PTPRO knockout mice [11]. Multiple signaling pathways are involved in the tumor suppressor function of PTPRO in HCC. Investigation on the correlation between PTPRO expression and STAT3 activity showed that PTPRO expression was negatively correlated with STAT3 activity in HCC tissues, indicating that PTPRO might suppress HCC via control of STAT3 activation. Indeed, PTPRO mediates STAT3 Y705 dephosphorylation via janus kinase 2 (JAK2) and S727 dephosphorylation via PI3K signaling [11]. In addition, it was reported that Toll-like receptor 4 (TLR4) activity protects against hepatocellular tumorigenesis and progression by regulating expression of DNA repair protein Ku70 in a mouse model [44]. PTPRO expression positively correlates with TLR4 expression in HCC specimens, and TLR4 expression and activity increased in PTPRO-overexpressing HCC cells stimulated with lipopolysaccharide (LPS). Further studies revealed that PTPRO regulates TLR4 via nuclear transcription factor-κB (NF-κB) activation, as the phosphorylation levels of IκBα and NF-κB/P65 increased in PTPRO-overexpressing HCC cells [45]. Further, PTPROt, a truncated isoform of PTPRO, was shown to play an important role in anti-tumor immunity in HCC microenvironment in a mouse model. PTPROt deficiency attenuated T cell-mediated anti-tumor immunity and remarkably promoted mouse HCC growth [46]. 

Protein tyrosine phosphatase receptor S (PTPRS), also known as PTPσ, was first identified in 1988 from a human placenta genomic DNA library [47]. The structure of PTPRS is composed of an extracellular region containing multiple Ig-like and fibronectin type III-like domains, a single transmembrane segment and two tandem intracytoplasmic catalytic domains. PTPRS was demonstrated to play an important role in the development of nervous system [48,49]. Recently, PTPRS was reported as tumor suppressor in a variety of cancers [29,50,51]. Mutation or deletion of PTPRS was detected in several human cancers including head and neck squamous-cell carcinoma [52], colorectal cancer [51], malignant melanoma [29], and cholangiocarcinoma [50]. Down-regulation of PTPRS was observed in HCC samples compared with non-tumor liver samples, and low expression of PTPRS was also observed in most of HCC cell lines [12]. Low expression of PTPRS was significantly associated with decreased overall survival and high risk of postoperative recurrence in HCC patients. PTPRS silencing promoted cell proliferation, migration and invasion both in vitro and in vivo. PTPRS could form a complex with epidermal growth factor receptor (EGFR) and regulate phosphorylation of EGFR at three tyrosine residues (Tyr 992, Tyr1045, and Tyr1068). HCC cell migration and invasion induced by PTPRS silencing can be inhibited by EGFR down-regulation, and overexpression of EGFR reversed the inhibition on cell migration and invasion induced by PTPRS silencing [12]. Both PI3K inhibitor and MEK1/2 inhibitor effectively inhibited the cell migration and invasion induced by enhanced EGFR phosphorylation, indicating EGFR/PI3K/MEK signaling was downstream of PTPRS in HCC. 

Protein tyrosine phosphatase receptor T (PTPRT), also known as PTPρ, is composed of an extracellular domain, a single transmembrane region containing a meprin-A5 antigen-PTP (MAM) domain, an Ig-like and four fibronectin type III-like repeats, and two tandem intracellular catalytic domains. Several evidences suggested that PTPRT functions as a tumor suppressor in human cancers including colorectal cancer [53], head and neck squamous cell carcinoma [54], and retinoblastoma [55]. HBxΔ127, a natural mutant of the hepatitis B virus (HBV) X protein (HBx) gene with COOH-terminal deletion from 382 to 401 bp, could remarkably increase the proliferation and migration of HCC cells compared with wild type HBx [56]. The mechanism underlying this phenomenon is that HBxΔ127 up-regulates miR-215 expression which down-regulates PTPRT protein expression in HCC cells [13]. Increased expression of PTPRT in HepG2 cells with HBxΔ127overexpression was able to decrease the tumor weight and volume in vivo [13]. Moreover, PTPRT is frequently mutated in human cancers such as colon, lung, skin and gastric cancer [53,57]. PTPRT with a mutation (A1022E) in the catalytic domain resulted in increased phosphorylation of STAT3 in head and neck squamous cell carcinoma (HNSCC) cells [54]. However, mutational analysis showed that PTPRT phosphatase domain mutations were not detected in HCC samples [57]. 

### 2.2. The Role of Classical Non-Receptor PTPs in Hepatocellular Carcinoma

PTP1B is encoded by the *PTPN1* gene. The structure of PTP1B is composed of an N-terminal catalytic domain, two proline-rich sequences, and a C-terminal hydrophobic region. PTP1B acts as a critical regulator in the pathogenesis such as diabetes and obesity [58,59]. Recently, pituitary homeobox 1 (PITX1) was reported to act as a tumor suppressor in hepatocarcinogenesis by activating the expression of p120RasGAP (also known as Ras p21 protein activator 1 (RASA1)), triggering Ras inactivation by converting GTP into GDP [60]. Downregulation of PITX1 mRNA and protein expression was frequently detected in the HCC patient samples with poorer prognosis [61]. PTP1B can prime PITX1 for proteasome-mediated degradation by directly dephosphorylating PITX1 at Y160, Y175, and Y179 residues, and decline of PITX1 reduced p120RasGAP transcriptional activity [14]. Through PITX1-p120RasGAP signaling axis, PTP1B possesses tumor promoting effects in HCC. Another study showed that PTP1B is recruited to MET receptor to reduce its phosphorylation by leukocyte cell-derived chemotaxin 2 (LECT2), and contributes to the blockage of vascular invasion and metastasis of HCC [15]. Zheng et al. showed that expression of PTP1B was low or lost in 65.7% HCC tumor tissues. Patients with lower PTP1B expression group had either shorter disease-free survival or worse overall survival [16], suggesting that PTP1B acts as a tumor suppressor in HCC. These studies indicated that PTP1B can be both oncogene and tumor suppressor depending on the substrates involved. In recent years, contrasting findings suggest that PTP1B plays two faces in other cancers too [62]. For example, PTP1B acts as a tumor suppressor in leukemia and lymphoma but can be as an oncogene in breast cancer and non-small cell lung cancer. It has been reported that PTP1B acts as an oncogene through interacting with several oncogenic substrates such as Src [63,64], ERK1/2 [65], p62dok [66,67], human epidermal growth factor receptor 2 (HER2) [68,69], and p130Cas [70]. Conversely, PTP1B can also act as a tumor suppressor by negatively regulating several oncogenic kinases such as Bcr-Abl [71], JAK-STAT [72,73], and β-catenin [74].

SHP-1, also known as HCP, HCPH, HPTP1C, PTP-1C, SHP-1L or SH-PTP1, is encoded by the *PTPN6* gene on chromosome 12. The SHP-1 protein contains two tandem Src homolog (SH2) domains which act as protein phospho-tyrosine binding domains, a catalytic protein tyrosine phosphatase (PTP) domain and a C-terminal tail [75]. SHP-1 has been reported as a tumor suppressor and a negative regulator of STAT3 in various cancer types [66,76,77,78]. The HCC cell lines with lower expression of SHP-1 showed higher expression of p-STAT3 Tyr705, and statistical analysis on HCC samples showed expression of SHP-1 had a negative correlation with p-STAT3 Tyr705 [21]. In transforming growth factor (TGF)-β1-induced STAT3 activated PLC5 cell model, SHP-1 overexpression strongly decreased the levels of p-STAT3 and depletion of SHP-1 increased levels of p-STAT3 [21]. Pyruvate kinase M2 (PKM2) was known to promote tumourigenesis through dimer formation of p-PKM2 Tyr105 [79,80]. Chen and colleagues confirmed that SHP-1 can directly dephosphorylate PKM2 at tyrosine Tyr105 to inhibit PKM2 activity in HCC, which is required for the proliferative function of PKM2 [81]. 

PTPN9, also called megakaryocyte protein tyrosine phosphatase 2 (PTP-MEG2), was cloned with sequence homologous to retinaldehyde-binding protein and yeast SEC14p [82]. The structure of PTPN9 contains an N-terminal domain that shares a significant similarity with yeast SEC14. High expression of PTPN9 was detected in brain, leukocytes and endocrine cells [83], and required for embryonic development [84], erythroid cell development [85,86], and intracellular secretary homotypic vesicle fusion in hematopoietic cells [87]. PTPN9 is also involved in numerous cellular processes including cell proliferation, differentiation and migration through the regulation of signaling pathways [88]. Dysfunction of PTPN9 causes a variety of human diseases such as cancer [89,90]. The expression level of PTPN9 was significantly reduced in HCC tumor tissues compared to non-tumorous tissues. PTPN9 expression was inversely associated with tumor size, and low expression of PTPN9 predicted poor survival in HCC patients [17]. Silencing of PTPN9 significantly reduced cell apoptosis and promoted cell proliferation in HCC cell line HepG2. Depletion of PTPN9 expression up-regulated tyrosine phosphorylation of STAT3 at Tyr705 site [17]. How PTPN9 affects tyrosine phosphorylation level of STAT3 in HCC still remains unclear. In breast cancer, PTPN9 indirectly inhibits activity of STAT3 and STAT5 through direct dephosphorylation of EGFR and HER2 [90]. PTPN9 also directly interacts with STAT3 and mediates its dephosphorylation in breast cancer cells [91]. The researches in breast cancer may provide some references for STAT3 regulation by PTPN9 in HCC. 

SHP-2, also known as PTPN11, was cloned in early 1990s as a non-receptor PTP [92]. The SHP-2 protein is composed of two SH2 domains which function as phospho-tyrosine binding domains and mediate the interaction of this PTP with its substrates, a catalytic protein-tyrosine phosphatase (PTPase) domain, and a C-terminal tail. SHP-2 has been considered as a proto-oncogene in several human cancers including leukemia [93], glioblastoma [94], gastric carcinoma [95,96], lung cancer [97], and breast cancer [98]. Somatic gain of-function mutations of *PTPN11* gene have been reported in about half of Noonan syndromes and certain types of leukemias [99,100]. However, SHP-2 does not just act as an oncogene, but also as a tumor suppressor in cancer progression. The higher protein expression of SHP-2 in colorectal cancer is related to better survival [101]. Recent studies demonstrated that SHP-2 may possess bi-directional functions in HCC. In a mouse model, hepatocyte-specific SHP-2 knockout resulted in regenerative hyperplasia and development of tumors [22], which may be due to compensatory hepatocyte proliferation triggered by inflammation [102], and increased synthesis of bile acid together with subsequent activation of Yap [103,104]. In contrast, Han et al. showed that SHP-2 silencing suppressed the proliferation of human HCC cells in vitro and inhibited the growth of HCC xenografts in vivo. Down-regulation of SHP-2 attenuated the adhesion and migration of HCC cells and diminished metastasized HCC formation in a mouse model. SHP-2 could promote HCC growth and metastasis by coordinately activating the Ras/Raf/Erk pathway and the PI3K/Akt/mTOR cascade [105]. SHP-2 sumoylation at lysine residue 590 may contribute to the activation of ERK signaling. Deng et al. recently reported that sumoylated SHP-2 facilitated the formation of SHP2-Gab1 complex to promote ERK activation [106]. SHP-2 increases β-catenin accumulation by inhibiting glycogen synthase kinase 3β (GSK3β)-mediated β-catenin degradation in liver cancer stem cells to enhance the self-renewal of liver cancer stem cells. Moreover, SHP-2 enhances β-catenin nuclear translocation via dephosphorylating CDC73 in liver cancer cells to promote the dedifferentiation of hepatoma cells, which finally promoted the expansion of cancer stem cells in cancer cell population [107]. In clinical samples, Jiang et al. reported decreased SHP-2 expression in tumor tissues compared with adjacent tissues and the positive rate was 66.1% and 96.7%, respectively. Further, lower SHP-2 expression in was significantly associated with shorter overall survival time in HCC [107], supporting SHP-2 as a tumor suppressor. However, Han et al. also examined HCC patient samples. They found elevated expression of SHP-2 in the majority of human HCC samples, and overexpression of SHP-2 correlated with advanced stage, poor differentiation and metastasis of HCC [105]. These opposite findings suggested the complexity of SHP-2 in HCC development. The role of SHP-2 in HCC needs to be further clarified.

PTPN12, also known as PTP-PEST, was first cloned from human skeletal muscle [108]. The structure of PTP N12 contains a C-terminal PEST motif which serves as a protein-protein interaction domain. PTPN12 regulates multiple oncogenic tyrosine kinases such as HER2 and EGFR [109], and is required for embryonic development [110]. PTPN12 regulates cell migration and cell-cell junctions through the interaction with cytoskeletal and signaling proteins [111,112]. PTPN12 was characterized as tumor suppressor in various human malignancies including breast cancer [109,113], colon cancer [112], ovarian cancer [114], nasopharyngeal carcinoma [115], and esophageal squamous cell carcinoma [116]. PTPN12 also plays an important role in the development of HCC. PTPN12 protein expression is frequently decreased or lost in human HCC tissues, and the decreased PTPN12 expression may represent an acquired recurrence phenotype of HCC [18]. Kodama and colleagues identified 233 candidate cancer genes using cell-based transposon mutagenesis screen assay, and the subsequent trunk driver analysis identified 23 candidate cancer genes that appear to function early in tumorigenesis. PTPN12 is one of these candidate cancer genes. The functional analysis showed that PTPN12 regulates epithelial-mesenchymal transition (EMT) in HCC cells, and down-regulation of PTPN12 also significantly increased the migration of HCC cell lines, SNU-387 and SNU-475 [117]. PTPN12-deficient mice displayed a failure of liver development and subsequent lethality [118], suggesting that PTPN12 may be critical for liver development as well. The decreased expression of PTPN12 and the mechanism underlying its inhibitory role towards migration and EMT in HCC still remain to be fully clarified. 

PTPN13, also known as protein tyrosine phosphatase-Basophil (PTP-BAS), human protein tyrosine phosphatase 1E (hPTP1E), PTPLE, protein-tyrosine phosphatase-L1 (PTPL1) or Fas-associated phosphatase 1 (FAP-1), was cloned independently by three groups [119,120,121]. The structure of PTPN13 is composed of a catalytic PTP domain at its C-terminus and two major structural domains: a region with five PDZ domains and a FERM domain that binds to plasma membrane and cytoskeletal elements. Accumulating evidences suggested that PTPN13 acts as a tumor suppressor in human cancers such as colorectal cancer [53], and breast cancer [122]. PTPN13 expression was often down-regulated or lost in HCC clinical samples and HCC cell lines. PTPN13 expression was positively correlated with overall survival, but negatively correlated with the cumulative recurrence rate [19,20]. The promoter of PTPN13 was detected hypermethylated with low PTPN13 expression [20], indicating that the promoter methylation may contribute to the down-regulation of PTPN13. HCC cell lines with low expression of PTPN13 demonstrated higher metastatic potential [19]. Overexpression of PTPN13 up-regulated the epithelial marker E-cadherin and down-regulated mesenchymal markers such as Snail, Slug and Matrix metallopeptidase 9 (MMP9) [19], suggesting PTPN13 can inhibit EMT in HCC progression. Overexpression of PTPN13 in HCC cell lines MHCC97H and HepG2 significantly reduced phosphorylation of ERK1/2 and STAT3 but not Src, indicating that PTPN13 may act as a tumor suppressor via ERK1/2 and/or STAT3 signaling in HCC.

### 2.3. The Role of Dual Specificity Phosphatases in Hepatocellular Carcinoma

The phosphatases of regenerating liver (PRL) belong to the dual-specificity superfamily. The PRL family includes three members PRL-1, PRL-2 and PRL-3. PRL-1, also known as PTP4A1, was originally identified as an immediate-early growth response gene in regenerating liver and mitogen-stimulated cells [123]. The protein structure of PRL-1 contains a PTP domain and a characteristic C-terminal prenylation motif. Overexpression of PRL-1 in NIH 3T3 cells resulted in altered cellular growth, enhanced cell proliferation, migration and invasion in vitro and enhanced cell metastasis in vivo [124,125,126,127]. Chinese hamster ovary (CHO) cells expressing PRL-1 developed metastatic tumors in mice [126]. Protein expression levels of PRL-1 were significantly higher in HCC tissues, and associated with more aggressive phenotype and poorer prognosis in HCC patients [24,25]. Overexpression of PRL-1 markedly up-regulated migration and invasion of HCC cells [25]. These findings suggested that PRL-1 acts as an oncogene in HCC. Jin and colleagues showed that PRL-1 regulated E-cadherin expression in HCC cells at both the mRNA and protein levels [25]. Loss of E-cadherin is correlated with tumor progression and metastasis in a variety of human cancers [128]. Further studies showed that PRL-1 can enhance phosphorylation of AKT at Ser474 and GSK3β at Ser9, which results in elevated levels of Snail expression and decreased E-cadherin expression [25], suggesting PRL-1 may regulate E-cadherin via PI3K/AKT/GSK3β pathway.

PRL-3, also known as PTP4A3, exhibits 76% of sequence identity with PRL-1 [110]. Like PRL-1, PRL-3 also displays oncogenic activity to promote cell proliferation, migration, invasion and metastasis [124]. PRL-3 was found to be overexpressed in many human cancers such as colorectal cancer [129], melanomas [130], breast cancer [131], gastric cancer [132], esophageal squamous cell carcinoma [133], acute myeloid leukemia [134], and ovarian [135]. Investigation on PRL-3 expression and its correlation with the clinical pathological features and prognosis in HCC by Mayinuer et al. showed that PRL-3 was up-regulated in patients with poorer differentiation, and higher expression of PRL-3 (both mRNA and protein) was significantly associated with poorer prognosis. Their studies also found that expression of PRL-3 in HCC patients was significantly correlated with the expression of several matrix metalloproteinases (MMPs) including MMP1, MMP9, MMP10 and MMP12 [26], which are key enzymes for invasion through the basement membrane and interstitial extracellular matrix [136]. The regulation of PRL-3 expression in physiological and pathological conditions involved many aspects such as transcription, translation and post-translation [137]. However, in HCC the molecular pathways involved in PRL-3 expression regulation and its downstream signaling still remain unclear. 

Dual-specificity phosphatase-1 (DUSP1), also known as MAP kinase phosphatase1 (MKP1), human VH1 phosphatase homolog (HVH1) or protein tyrosine phosphatase non-receptor 10 (PTPN10), belongs to a family of phosphatases with dual specificity for threonine and tyrosine, which was first found in cultured murine cells [138]. The structure of DUSP1 is composed of an N-terminal non-catalytic domain and a C-terminal catalytic domain. DUSP1 plays an important role in cell proliferation, cycle arrest, differentiation, transformation and apoptosis [139], and is involved in development and progression of many cancers such as prostatic cancer [140], pancreatic cancer [141], lung cancer [142], breast cancer [143] and gastric cancer [144], head and neck squamous cell carcinoma [145] and gallbladder cancer [146]. The mechanism and function of DUSP1 in cancers varies dependent on the cancer type. DUSP1 can promote carcinogenesis of prostatic cancer [140], pancreatic cancer [141], lung cancer [142], breast cancer [143] and gastric cancer [144]. The mechanism underlying the oncogenic function of DUSP1 in these cancers is probably due to DUSP1-medaited inhibition of JNK activation and subsequent escape from JNK-induced apoptosis of the cancer cells. In head and neck squamous cell carcinoma, DUSP1 suppresses carcinogenesis via decreasing IL-1β expression in tumor pro-inflammatory microenvironment [145]. In gallbladder cancer, DUSP1 also inhibits cancer cell growth and metastasis via targeting the DUSP1-pERK-MMP2/VEGF signaling pathway [146]. In human HCC, the mRNA levels of DUSP1 were found significantly higher in tumors with better prognosis even compared with normal or non-tumorous surrounding tissues [27]. Sharply declined DUSP1 protein expression was observed in all HCC patient samples with poorer prognosis [27] and Tsujita et al. showed that patients with positive staining for DUSP1 have a longer survival [147]. In rat models, Fisher 344 (F344) rats with lower DUSP1 were susceptible to HCC development, while Brown Norway (BN) rats with higher DUSP1 were resistant HCC development [28,148]. These findings suggested that DUSP1 could suppress carcinogenesis in HCC. Up-regulation of active ERK1/2 was detected in HCC tissues, and the highest levels of ERK1/2 appeared in patient samples with poorer prognosis [27]. In rat modes, declined DUSP1 expression and elevated ERK activation were observed in susceptible F344 rats compared with resistant BN rats [28]. DUSP1 reactivation led to suppression of ERK activity, inhibition of proliferation and induction of apoptosis in human HCC cell lines [27]. A reciprocal regulation between ERK and DUSP1 was discovered. DUSP1 dephosphorylates ERK to inactivate it [149], while active ERK phosphorylates DUSP1 at ser296, which facilitates its ubiquitination and proteasomal degradation [150,151]. Declined DUSP1 expression may interrupt the positive regulatory loop between ERK and DUSP1 and promote development and progression of HCC. 

## 3. Targeting the PTPs for Therapy in Hepatocellular Carcinoma

HCC is one of the most lethal cancers. Many drugs were developed in clinical to therapy HCC, but to date the therapy to HCC continues to pose a challenge, in part because of the poor chemosensitivity or chemoresistance [152]. As described above, PTPs plays an important role on the formation and development of HCC. Targeting PTPs for the therapy to HCC may provide an alternative choice. The strategy of targeting PTPs should depend on its function on HCC. To PTPs as tumor suppressor, it is a anticancer strategy to develop agonists to activate these PTPs activity or increase these PTPs expression. To PTPs as tumor promoter, it is an anticancer strategy to develop inhibitors to inhibit these PTPs activity.

STAT3 is a key regulator of inflammation, cell survival, and tumorigenesis in liver cells [153]. It was reported that nearly 60% of clinical HCC tumor samples showed nuclear phosphorylated-STAT3 staining [154], suggesting that targeting STAT3 signaling pathway may be an effective treatment for HCC with constitutively or inductively active STAT3. Sorafenib was originally developed to inhibit the Ras-Raf-MEK1/2-ERK1/2 signaling pathway by specifically targeting Raf-1 kinase [155]. But sorafenib and its derivatives were found to inhibit tumor growth via a kinase-independent mechanism in HCC. Sorafenib and its derivatives can form a salt bridge with the D61 residue of SHP-1 in the N-SH2 domain, which releases the auto-inhibition of SHP-1 and activates SHP-1 [153]. Accumulating data showed that sorafenib and its derivatives can effectively suppress tumor of HCC through SHP1/STAT3 axis [21,75,156,157,158,159,160]. Some sorafenib derivatives displayed more potent anti-HCC activity than sorafenib, even for sorafenib-resistant HCC cells [157,158]. 

Other kinase inhibitors, such as nintedanib [161], regorafenib [162], and dovitinib [163], induced significant anti-HCC activity independent of kinase inhibition activity by relieving auto-inhibition of SHP-1 to activate its activity. Increasing the transcription of SHP-1 is another approach to suppress STAT3 signaling for anti-HCC therapy. SC-2001, an obatoclax analogue, showed potential antitumor effect on HCC cells [164,165,166]. The mechanism of SHP-1 activation by SC-2001 is that it induced Regulatory factor X-1 (RFX-1)-1 to bind to the SHP-1 promoter, and activated SHP-1 transcription [164]. Transcription of SHP-1 is activated by other small molecules, such as emodin [167], evodiamine [168], honokiol [169], and TFPA [170], from natural plants. These molecules also displayed effective anti-HCC activity. In addition to SHP1, several other PTPs such as PTPRD, PTPRO, PTPN9, and PTPN13, also dephosphorylate STAT3 at tyrosine 705 to reduce STAT3 activity in HCC cells [7,11,17,19]. Development of some strategies to activate their activity or increase their transcription might provide new therapeutic strategy for HCC patients. 

The combination of SHP-1 agonists and conventional therapies, such as radiation therapy or chemical therapy, can help improve the effectiveness of traditional therapies. Dovitinib, a SHP-1 agonist, enhanced the effect of radiation therapy against HCC in vitro and in vivo [171]. The combination of dovitinib and tigatuzumab restored the sensitivity of HCC cells to TRAIL (tumor necrosis factor-related apoptosis-inducing ligand)- and tigatuzumab-induced apoptosis [172]. The combination of sorafenib and SC-43 decreased tumor size and prolonged survival in mouse model [173]. The combination of SC-2001 and sorafenib strongly inhibited tumor growth in both wild-type and sorafenib-resistant HCC cell bearing xenograft models [165]. SHP-1 agonist SC-59 displays a better synergistic effect when used in combination with radiotherapy for the treatment of HCC [174]. SHP-2 silencing by siRNA increased the sensitivity of hepatoma cells to sorafenib [105], suggesting that combined use of SHP-2 inhibitor with sorafenib might improve anti-HCC therapeutic efficacy.

Moreover, Baburajeev et al. reported that a synthesized small molecule inhibitor of PTP1B, 6-(2-benzylphenyl)-3-phenyl-[1,2,4]triazolo[3][1,3,4]thiadiazole (BPTT), showed effective inhibition on cell invasion and tumor volume of HCC [175]. In addition, sorafenib can up-regulate PITX-p120RasGAP axis via inhibition of PTP1B [14], indicating inhibition of PTP1B may be effective for the treatment of HCC. 

## 4. Conclusions 

Here we have illustrated that PTPs play a growing important role in HCC progression via regulation of cell proliferation, migration and invasion. Different strategies need to be employed to target different types of PTPs. Among the 16 reported PTPs involved in HCC, most of them act as tumor suppressor, e.g., PTPRD, PTPRF, PTPRH, PTPRO, PTPRS, PTPRT, PTPN9, PTPNN12, PTPN13, SHP-1 and DUSP1. Although developing small molecule agonists of proteins are much more difficult than developing small molecule inhibitors, it is not impossible. Several agonists against SHP-1 showed effective suppression towards HCC tumor. Moreover, expression of some PTP tumor suppressors, such as PTPRD, PTPRO, PTPN12, PTPN13 and DUSP1, is reduced due to epigenetic silencing (Table 2). Restoring these PTPs expression by demethylating agents can also be an activation strategy. On the contrary, both PRL1 and PRL3, displayed oncogenic activities in HCC making the blockade of their activity through developing small molecule inhibitors a potential therapy for HCC. Last, SHP2 and PTP1B displayed both oncogenic and tumor suppressive activities in HCC. Therefore, extra caution need to be taken when targeting SHP2 and PTP1B in HCC. 

At present, the 16 reported PTPs involved in HCC regulation all belong to the Class I family of PTP. The effect of Class II, Class III and Class IV PTPs on HCC are not clear. To fully understand the roles of PTPs in HCC, it is necessary to carry out a thorough investigation of the expression and mutation of tyrosine phosphatome in HCC samples, in order to fully identify critical PTPs in HCC. In addition, the dissection of the key molecular signaling pathways underlying the functions of these critical PTPs need to be understood. The improvement of our knowledge on PTPs and the relevant pathways may shed light in the development of new anti-cancer reagent for HCC. 

## Figures and Tables

**Table 1:** Protein tyrosine phosphatases (PTPs) discussed in this review with their potential role in hepatocellular carcinoma.

PTPs		Observations	TSG or Onco	Ref.
Receptor PTPs	PTPRD	PTPRD expression was downregulated or loss; High expression of PTPRD has a long-term survival rate and less chance of recurrent liver cancer.	TSG	[7,8]
	PTPRF	PTPRF was frequent downregulated in most of HCC patients, and upregulation of PTPRF associated with better prognosis. PTPRF suppressed cell proliferation, colony formation in vitro and inhibited tumor growth in vivo.	TSG	[9]
	PTPRH	PTPRH expression in moderately differentiated HCCs and in all poorly differentiated HCCs was greatly reduced or loss. Overexpression of PTPRH reduce both migratory activity and growth rate of cells	TSG	[10]
	PTPRO	PTPRO expression was significantly reduced in human HCC specimens. Overexpression of PTPRO promoted apoptosis i and nhibited proliferation in vitro, and tumor size and number were increased in PTPRO knockout mice.	TSG	[11]
	PTPRS	Downregulation of PTPRS was observed in HCC cell lines and samples, and significantly associated with decreased overall survival and high risk of recurrence. PTPRS silencing promoted cell proliferation, migration and invasion both in vitro and in vivo.	TSG	[12]
	PTPRT	Increased expression of PTPRT in HepG2-xΔ127 cells treated decreased the tumor weight and volume in vivo	TSG	[13]
non-receptor PTPs	PTP1B	PTP1B prime protein degradation of PITX1 to reduce p120RasGAP transcription.	Onco	[14]
		Patients with low PTP1B expression had either shorter disease-free survival or worse overall survival. PTP1B could reduce phosphorylation of MET receptor to block vascular invasion and metastasis.	TSG	[15,16]
	PTPN9	PTPN9 expression was significantly reduced in tumor tissues, and low expression of PTPN9 predicted poor survival. PTPN9 silencing reduced apoptosis and promoted proliferation in HepG2 cells.	TSG	[17]
	PTPN12	PTPN12 expression is frequently decreased or loss in HCC tissues. down-regulation of PTPN12 also significantly increased the migration of HCC cell lines.	TSG	[18]
	PTPN13	PTPN13 expression was often downregulated or loss in HCC tissues and HCC cell lines, and positively correlated with overall survival but negatively correlated with the recurrence rate. Overexpression of PTPN13 inhibit EMT in HCC progression.	TSG	[19,20]
	SHP-1	SHP-1 expression has a negative correlation with p-STAT3 Tyr705 in HCC, and SHP-1 overexpression abolished TGF-β1-induced p-STAT3 Tyr705.	TSG	[21]
	SHP-2	SHP2 knockout mice result in development of tumors and enhance diethylenenitrite (DEN)-induced HCC development.	Onco	[22]
		low Shp2 expression was significantly associated with short overall survival time. SHP2 could promote HCC cell growth and metastasis by coordinately activating the Ras/Raf/Erk pathway and the PI3-K/Akt/mTOR cascade.	TSG	[23]
DUSPs	PRL1	High PRL-1 expression was associated with more aggressive phenotype and poorer prognosis in HCC patients. Overpression of PRL-1 markedly enhanced migration and invasion of HCC cells.	Onco	[24,25]
	PRL-3	PRL-3 expression (both mRNA and protein) was significantly associated with poor differentiation and prognosis, and positive correlation with matrix metalloproteinase MMP1, MMP9, MMP10 and MMP12.	Onco	[26]
	DUSP1	High DUSP1 expression was associated with better prognosis. In rat models, low DUSP1 expression was more susceptible to develop HCC.	TSG	[27,28]

* TSG: Tumor suppressor gene; Onco: Oncogene; PTPRD: protein tyrosine phosphatase receptor delta; PTPRF: protein tyrosine phosphatase receptor type F; PTPRH: protein tyrosine phosphatase receptor type H; PTPRO: protein tyrosine phosphatase receptor type O; PTPRS: protein tyrosine phosphatase receptor S; PTPRT: protein tyrosine phosphatase receptor T; PTP1B: protein tyrosine phosphatase 1B; PTPN: protein tyrosine phosphatase, non-receptor; SHP: Src homology 2-containing phosphotyrosine phosphatase; PRL: phosphatase of regenerating liver; DUSPs: dual specificity phosphatases; HCC: hepatocellular carcinoma; MET: mesenchymal-epithelial transition factor; STAT3: signal transducer and activator of transcription 3; Tyr705: tyrosine 705; TGF: transforming growth factor; Ras/Raf/Erk:rat sarcoma/ rapidly accelerating fibrosarcoma/ extracellular signal-regulated kinase; PI3-K/Akt/mTOR: phosphotidylinsitol-3-OH kinase/ alpha serine/threonine-protein kinase/mechanistic target of rapamycin.

**Table 2:** The mechanisms responsible for regulation of PTPs in HCC.

PTPs	Mechanisms for PTPs Regulation in HCC
PTPRD	Epigenetic silencing is partly responsible for the reduction of PTPRD expression [8]. Deletion and mutation of PTPRD gene are also identified in HCC cell lines and tumor tissues [30,36], miR-135a-5p targeting PTPRD mRNA impairs PTPRD expression [7].
PTPRF	NA
PTPRH	NA
PTPRO	Epigenetic silencing is responsible for the reduction of PTPRO expression [43].
PTPRS	Epigenetic silencing is responsible for the reduction of PTPRS expression [12].
PTPRT	miR-215 targets PTPRT and down-regulates its protein expression in HepG2 cells [13]
PTP1B	NA
SHP-1	NA
PTPN9	NA
SHP-2	SHP-2 activation may be due to protein SUMOylation at lysine residue 590 [106].
PTPN12	NA
PTPN13	Epigenetic silencing is responsible for the reduction of PTPN13 expression [20].
PRL-1	Copy number amplification may be responsible for the increase expression of PRL-1 [25]
PRL-3	NA
DUSP1	DUSP1 inactivation is due to ubiquitination or promoter hypermethylation associated with loss of heterozygosity at the DUSP1 locus [27].

NA = Not Available.
